# Department of Therapeutics

**Published:** 1894-03

**Authors:** 

**Affiliations:** Demonstrator of Physiology and Lecturer on the History of Medicine in the Medical Department of the University of Texas, Etc.; Galveston


### Current Medical Literature.

### DEPARTMENT OF THERAPEUTICS.

EDITED BY DAVID CERNA, M. D., PH. D.,
Demonstrator of Physiology and Lecturer on the History of Medicine in the Medical Department of the University of Texas, etc.

THE USE OF ERGOT IN LABOR.—From a clinical study of the uses of ergot in labor, Dr. R. B. McCall, of Hammersville, Ohio (*American Medico-Surgical Bulletin*, Feb. 1, 1894), draws the following conclusions: 1. That ergot is a true oxytocic by virtue of its unaided power exercised on the uterine center of the medulla spinalis, to excite the gravid uterus to contract on its contents. 2. That in all cases of hemorrhage from non-gravid relaxed uterus, ergot in suitable dosage compels firm, persistent contraction. 3. That, in view of the two facts last named, its employment as a safeguard after delivery of the placenta is conservative and practical, and devoid of danger. The author also believes that the drug possesses some value as a hemostatic.

---

THE MEDICINAL TREATMENT OF TYPHLITIS.—The *Revue Générale de Clinique et de Thérapeutique* for December 6th, gives a summary of M. Grasset's views on this subject. The therapeutical indication, he says, varies accordingly as there is an actual acute inflammatory attack, or as the case is one of recurrent typhlitis between the attacks, or as there is typhlitis with persistent cœcal engorgement. In the last named case, medicinal treatment usually fails, and an appeal to the knife becomes a necessity. In the case of a recent typhlitis with acute exacerbations, the physician may treat the appendicular colic, combatting the pain with warm baths, evacuating the intestinal contents, and, if possible, resolving the inflammation. M. Grasset fulfills these various indications as follows: 1. He gives a full warm bath of from half an hour to an hour in duration. 2. Every hour, the patient is to take a teaspoonful of a purgative potion, consisting of one part each of castor oil and oil of sweet almonds and two parts of syrup of lemon, until a copious evacuation of the bowels has been produced. 3. The iliac region is to be annointed with an ointment consisting of mercurial ointment and belladonna, and this is to be followed by the application of a large, hot, thin poultice of linseed meal. In very stubborn cases, M. Grasset adds to the purgative potion a drop of croton oil. In cases of recurrent typhlitis, the treatment during the interval consists in preventing overloading of the intestinal canal, and in the use of antiseptics, revulsives, and resolvents to the seat of inflammation. For this purpose he advises: 1. A diet that leaves little residue. 2. Every week the application of the actual cautery to the painful and infiltrated region: also every day or as often as may be necessary, frictions with an ointment containing belladonna. 3. To overcome constipation, at bedtime a a laxative pill, consisting of one-sixth of a grain each of powdered belladonna and podophyllin. 4. The insurance of intestinal antisepsis by taking before and after each meal a capsule containing seven or eight grains of benzo-naphthol.—*New York Medical Journal*, January 27, 1894.

---

SULPHATE OF COPPER IN THE TREATMENT OF SYPHILIS.—Dr. A. F. Price, of the United States Navy (*Medical Record*, February 3, 1894,) gives his experience in the treatment of syphilis with sulphate of copper. He has employed the drug in various stages of the disease, and the results obtained lead the author to formulate the following conclusions: 1. Copper exercises a specific action in syphilis, which is especially directed toward the lymphatic system. It is, for this reason, more radically curative than mercury. 2. It is slow in removing the skin symptoms of the secondary stage. 3. It prevents the development of mucous patches and throat symptoms. 4. It is a very active drug, and it is wise to omit its use one day in a week, and sometimes more frequently. The signs of its excessive and injurious action are first a voracious appetite, and this is rapidly followed, if the dose is not reduced or the drug temporarily discontinued, by prostration, giddiness, pallor, and a rapid and weak pulse. 5. The average dose of sulphate of copper is $\frac{1}{30}$ of a grain thrice daily. It is better to give it with the sulphate of iron. It can be given either in pill or solution. 6. This dose is absolutely dangerous in cases of syphilitic cachexia. It produces at once excessive and alarming prostration. If a sufficiently small dose of the drug is given at first, a tolerance of it is gradually established, so that the average dose may in time be obtained. The author is inclined to think that in some cachectic cases as small a dose as the 1-10000 of a grain may be necessary, given once daily. The use of iron, arsenic, and iodide is also usually necessary in old syphilis.

---

NITRATE OF ACONITINE IN FACIAL ERYSIPELAS.—Dr. Tison has used this remedy since 1888, in every case of erysipelas which has come under his treatment. The results have been highly satisfactory. Pain and other symptoms have been rapidly subdued, save in two instances, and in neither of these could the complications which arose be due to the treatment. Although nitrate of aconitine is highly poisonous, if used as suggested by the author, the danger of untoward symptoms is very slight. The alkaloid is dissolved in a mixture of glycerine, alcohol, and distilled water, the mixture having the same specific gravity as water, and containing one milligramme in fifty minims. Only two to four milligrammes are required in any one case. The method of procedure is as follows: According to the condition of the stomach, either an emetic or a purgative is administered, immediately after which nitrate of aconitine is given in a mixture containing one milligramme for the twenty-four hours' dosage. The affected part is painted with a saturated ethereal solution of camphor, which acts as an excellent sedative. When all signs of inflammation have subsided, the part is washed with a solution of lysol.—*La Semaine Médicale—Medical Record*, February 3, 1894.

---

THE USES OF CATARTHINIC ACID.—This acid has been obtained from senna by M. Gentz (*Vratch*, No. 30—*Nouveaux Remèdes*, November 24, 1893—*Journal de Médecine de Paris*, December 17, 1893). It appears in the form of a brown powder, little soluble in water. This purgative may be given in the dose of five to fifteen centigrammes, and its effects are manifested eight to ten hours after administration. On healthy subjects, having taken the remedy solely to study its physiological action, it causes frequent stools (about five in half a day) and mild colic. In persons affected with chronic constipation, on the contrary, colic is not observed in the generality of the cases. The action of the medicine is more slow and less attended with disagreeable sensations. Thanks to this latter circumstance, and taking into consideration the absence of all disagreeable taste, and the certainty and energy of its action, the author was able to predict for the catarthinic acid an honorable place among the purgatives. In infants of two to four years the acid was prescribed in a dose of 0.05 gramme mixed with sugar, and a dose of 0.15 gramme for adults. Dehio tried the medicine on five well persons, and five persons suffering from chronic constipation. He offers the following formula:

℞—Catarthinic acid.. .....0.05 to 0.15 gramme.
White sugar...........0.30 to 0.50 gramme.
M. For *one* powder. Of such a powder take one every hour or two.

Dehio has not yet had occasion to study the action of the catarthinic acid for a long time, but considering the results of his ten trials, he is much inclined to hope that in these cases the remedy may render signal service.

---

GUAIACOL AS AN ANTIPYRETIC.—The *Medical News* for January 27th (*N. Y. Medical Journal*, February 10th, 1894), publishes a portion of a lecture delivered at the Pennsylvania Hospital, on January 13th, by Dr. J. M. Da Costa, of Philadelphia, entitled Clinical Remarks on the External Use of Guaiacol in reducing High Temperature in Typhoid Fever and othee Febrile Diseases. Dr. Da Costa thinks that the action of guaiacol is somewhat inferior to that of the cold bath as regards promptness, but he has observed that the reduction of temperature which is produced is more lasting. He thinks it preferable to the use of cold baths, particularly in cases where proper appliances for administering the baths are wanting, and where, from the nature of the case, it is particularly objectionable to move the patient. The odor of guaiacol is an objection to its use, and this Dr. Da Costa has not been able to overcome. Oil of bergamot has been tried, but the odor of guaiacol overcomes that of the bergamot. Cologne and oil of sassafras also have been tried, but the best result has been obtained with oil of cloves. Guaiacol is to be rubbed upon the skin of the abdomen, or thigh, with a camel's hair brush, over a space previously washed with soap and water. The largest amount that has been applied at once, in Dr. Da Costa's experience, is sixty drops, but from the application of fifty drops he has witnessed effects that cause him to advise that rarely should so large a dose be used. Thirty drops he thinks about the average dose. The guaiacol is to be rubbed in slowly, and the surface to which it is applied need not be uncovered, for the application can be made under the bed clothing, and it is well to cover the surface with a piece of lint and with wax paper. The dose should be proportionate to the height of the fever; with a temperature of 103° F., it should not exceed twenty minims at the first trial. It is not necessary to rub the guaiacol in, but if friction is not used the effect is neither so rapid nor so complete. The quickest way is to paint the guaiacol on the surface and then rub it in with the hand. Five minutes are enough for the purpose. The sensation, which is not unpleasant, is likened to that produced by the application of menthol. Dr. Da Costa thinks that guaiacol is absorbed by the skin, and that it is carried by the circulation to the heat cestres, upon which it acts as an antithermic. Apparently its action does not involve the depression that follows the use of antipyrin, phenacetin, and other remedies, belonging to the coal-tar products. Moreover, it produces less sweating. In no instance, under Dr. Da Costa's observation, was there any albumen in the urine, or any sign of kidney irritation detected that could be imputed to the use of guaiacol; nevertheless, he advises close examination of the urine in all cases in which the remedy is used.

